# Surgical Versus Nonsurgical Management of Acetabular Fractures With Associated Patterns in Elderly Patients: Factors Affecting Outcomes

**DOI:** 10.5435/JAAOSGlobal-D-22-00014

**Published:** 2022-03-02

**Authors:** Isabella M. Heimke, Nicholas R. Scarcella, Natasha M. Simske, Ryan Furdock, Heather A. Vallier

**Affiliations:** From the Department of Orthopaedic Surgery, MetroHealth Medical Center affiliated with Case Western Reserve University, Cleveland, OH.

## Abstract

Among elderly patients, anterior column posterior hemitransverse (ACPHT) and associated both column (ABC) are common acetabular fracture patterns after low-energy mechanisms. Given the paucity of outcomes data in this cohort, the goal of this study was to determine the favorability of results with surgical versus nonsurgical management. Secondarily, factors linked with poor functional outcomes were assessed.

**Methods:**

Over a 16-year period, 81 patients aged ≥60 years with 82 ACPHT and ABC acetabular fractures were evaluated. Retrospectively, patient demographics, injury details, and early and late complications were collected. Functional outcomes were assessed with the Musculoskeletal Function Assessment (MFA) after a minimum of 12 months of follow-up.

**Results:**

During the study period, 81 patients sustained 82 ACPHT (n = 35, 43%) or ABC (n = 47) fractures, most secondary to low-energy falls (71%). Patients managed surgically were younger, had higher-energy mechanisms, and more often had an associated hip dislocation or marginal impaction (all *P* < 0.05). Of note, 42.3% and 18.5% of patients had early and late complications, respectively, with no differences between surgical and nonsurgical groups. Posttraumatic arthrosis (PTA) was noted in 27% overall (36% surgical versus 16% nonsurgical, *P* = 0.10). The mean MFA score was 25.2 after 59 months. Better outcomes were associated with high-energy mechanisms, multiple injuries, and surgical management (all *P* < 0.05). The worst MFA outcomes were among patients with PTA (40.2) and those requiring a secondary procedure (45.7), both *P* < 0.05.

**Discussion:**

Nonsurgical management had a low rate of PTA. Mitigating PTA and decreasing the rate of secondary surgeries seem crucial achieving satisfactory outcomes. Higher-energy injuries benefit from open reduction and internal fixation, as indicated by better MFA scores.

Acetabular fractures are a challenging issue in the elderly population because of underlying comorbidities, poor bone quality, and baseline functional limitations, which are common in this group.^1^ As our population ages and demonstrates increased longevity^2-4^ and as treatment techniques and capabilities evolve, there is a need to assess which treatments mitigate complications and improve outcomes. Goals of return to previous lifestyle and functionality are essential.^5^ The most frequent mechanism for an acetabular fracture in this population is a low-energy fall onto the hip.^6,7^ Even with minimal force, severe articular injury and fracture displacement may occur due to low bone density. This coupled with common age-related medical and social comorbidities make such injuries more complex to treat.^8^ Anterior column post hemitransverse (ACPHT) and associated both column (ABC) fracture patterns are common in the elderly,^9^ often resulting from lateral force directed onto the greater trochanter at the time of impact. Many of these patterns may be treated surgically or nonsurgically. Although relative indications for surgical and nonsurgical treatments exist, variability in recommendations and treatment decisions depends on the patient and the surgeon.

Few previous studies exist, relating such acetabular fractures and treatment to functional outcomes. However, a study by Clarke-Jenssen et al^10^ found that nonsurgical management of these borderline fractures has produced functional clinical results, but this study did not examine patient-reported outcomes of these patients. Although studies have shown that surgical treatment may be associated with better functional outcomes,^11,12^ elderly patients may not be suited for surgery due to comorbidities that render them poor surgical candidates and necessitate conservative management. In contrast to proximal femoral (hip) fractures, which are nearly always treated surgically to promote immediate weight bearing and mobility from bed, most acetabular fractures require protected weight bearing after fixation. Therefore, surgical intervention cannot be used under these circumstances to optimize weight-bearing status postinjury.

For elderly patients with ACPHT and ABC acetabular fracture patterns, numerous factors influence treatment including associated injuries, age, and mechanism of injury. The objective of this study was to determine whether surgical or nonsurgical treatment of ACPHT and ABC fractures patients aged 60 years and older results in optimal results and outcomes. A secondary objective was to identify factors associated with poor functional outcomes.

## Methods

A retrospective study was conducted at a level 1 trauma center to assess 982 skeletally mature patients treated for acetabular fracture over 16 years. All 81 patients with 82 ACPHT or ABC fractures and age at least 60 years at the time of injury were included. To assess short-term complications, 71 patients with 72 fractures had a minimum of 12-week follow-up postinjury or sustained early complications before 12 weeks. To assess long-term complications and additional surgeries, 41 patients with 42 fractures had a minimum 12-month follow-up or long-term complication or secondary surgery before 12 months. Associated injuries to the head, spine, upper extremities, lower extremities, pelvis, chest, and abdomen were recorded. Early complications included superficial infection, deep infection requiring débridement in the operating room, deep vein thrombosis proximal to the knee, pulmonary embolism, and iatrogenic nerve injury. Late complications included PTA, osteonecrosis, and heterotopic ossification and were evaluated at 12 months or before 12 months if symptomatic. Additional unplanned surgeries were also recorded.

Fellowship-trained orthopaedic trauma surgeons classified fracture patterns using the Letournel and Judet classification^16^ and developed a treatment plan with each patient. After a minimum of 12 months of follow-up, patients completed a functional outcome survey (Musculoskeletal Function Assessment [MFA]). The MFA is a generalized functional outcome survey, which is valid, reliable, and responsive for trauma patients. Patients were contacted a minimum of three times by phone and twice by mail to complete surveys by trained research staff. Scores for the MFA range from 0 to 100, with low scores equating to a good functional outcome and high scores with a poor functional outcome.^13-15^

Clinical outcome variables included early or late complications and the need for secondary reconstructive procedures. Bivariate analysis with a Fisher exact test was used to assess associations between the possible predictive variables and clinical outcomes. The variables included fracture pattern and features and demographic and medical variables. The Student *t*-test was used to determine correlations between functional outcomes and treatment characteristics. Statistical significance was set at *P* < 0.05.

## Results

### Demographics and Injury Features

The study group included 81 patients ranging from 60 to 94 years of age (mean 72.7 years of age), sustaining 82 ACPHT (n = 35 (42.7%)) or ABC (n = 47) fractures to the acetabulum. The most common mechanism was a fall, occurring in 81.5%, most of which were falls from standing height. The mean Injury Severity Score for all patients was 14.1, and 26 patients (32.1%) had isolated injuries. Half of the fractures were treated surgically (40 patients with 41 fractures). Table 1 provides comparison of the two treatment groups.

**Table 1:** Demographic and Injury Features Are Shown for Patients Treated Surgically Versus Nonsurgically

Demographics and Injury Features	Nonsurgical (n = 41 Patients With 41 Fractures)	ORIF (n = 40 Patients With 41 Fractures)	All Patients	*P* Value
Male	30 (73.2%)	30 (75.0%)	60 (74.1%)	
Mean age (yrs)	74.8	70.5	72.7	0.045
Age range	60-90	60-94	60-94	
Median age (yrs)	75	66	72	
Mean BMI, kg/m^2^	26.6	27.9	24.5	0.27
Mean ISS	11.9	16.5	14.1	0.002
Mechanism of injury, n (%)				0.004
Bicycle	0	2 (5.0)	2 (2.5)	
Fall from height	6 (14.6)	13 (32.5)	19 (23.5)	
Fall from stand	29 (70.7)	18 (45.0)	47 (58.0)	
MVC	4 (9.8)	6 (15.0)	10 (12.4)	
Pedestrian versus vehicle	2 (4.9)	1 (2.5)	3 (3.7)	
Fracture pattern, n (%)				0.045
ABC	28 (68.3)	19 (46.3)	47 (57.3)	
ACPHT	13 (31.7)	22 (53.7)	35 (42.7)	
Associated injuries, n (%)	28 (68.3)	27 (67.5)	55 (67.9)	0.89
Isolated injuries, n (%)	13 (31.7)	13 (32.5)	26 (32.1)	
Injury features, n (%)				
Hip dislocation	2 (4.9)	5 (12.2)	7 (8.5)	0.001
Femoral head injury	0	1 (2.4)	1 (1.2)	
Marginal impaction	2 (4.9)	8 (19.5)	10 (12.2)	0.0005

ABC = associated both column, ACPHT = anterior column posterior hemitransverse, BMI = body mass index, ISS = Injury Severity Score, MVC = motor vehicle collision

The groups were similar in sex (74% male overall) and BMI (24.5 kg/m^2^ overall), although the patients treated surgically were slightly younger (mean 70.5 versus 74.8 years, *P* = 0.045). Patients treated surgically were more likely to have a high-energy mechanism (55.0% versus 29.3%, *P* = 0.004) and thus a higher Injury Severity Score (16.5 versus 11.9, *P* = 0.002). Consistent with this, patients treated surgically were more likely to have central hip dislocation at the time of injury (12.2% versus 4.9%, *P* = 0.001) and to have marginal impaction of their acetabular articular surface (19.5% versus 4.9%, *P* = 0.0005). Surgical patients had longer median hospital stay (8.5 versus 4.0 days, *P* = 0.0006).

### Early and Late Complications

Mean clinical follow-up was 23.4 months. Thirty patients (42.3%) had an early complication, with 14 (35%) in surgically treated patients and 16 (39%) in nonsurgically treated patients (Table 2). Infections were infrequent, occurring in 2 patients, whereas thrombotic complications occurred in 9 patients after nonsurgical care versus 5 after surgery (not significant). Fifteen patients developed late complications, with 10 (25%) after open reduction and internal fixation (ORIF), including 8 of those 10 with PTA. Five (12.2%) nonsurgically treated patients had late complications, three of these with PTA. Secondary surgeries mainly consisted of total hip arthroplasty (THA) for pain relief, including 6 after ORIF and 2 after nonsurgical management. The most common long-term complication was PTA, occurring in 11 patients (26.8%), with 8 patients treated surgically (36.4%), and 3 treated nonsurgically (15.8%), *P* = 0.096. Arthrosis was associated with initial injury features, primarily marginal impaction; all 10 patients with marginal impaction developed PTA. Central dislocation was also more likely to be associated with PTA. Three of the five patients treated with ORIF and developing PTA had a dislocation, and both of the nonsurgical patients with dislocation also developed PTA.

**Table 2:** Complications and Hospital Course Are Presented Based on the Type of Treatment

Complications and Hospital Course	Nonsurgical (n = 41)	ORIF (n = 40)	All Patients	*P* Value
Wound infection, n (%)	0	2 (4.9)	2 (2.4)	0.15
DVT, n (%)	7 (17.1)	4 (10.0)	11 (13.6)	0.86
PE, n (%)	2 (4.9)	1 (2.5)	3 (3.7)	0.57
Pneumonia, n (%)	3 (7.3)	4 (10.0)	7 (8.6)	0.67
UTI, n (%)	7 (17.1)	5 (12.5)	12 (14.8)	0.56
Median hospital stay (d)	4 (1-29)	8.5 (4-25)	8 (1-29)	0.0006
Mortality (all), n (%)	20 (48.8)	12 (30.0)	32 (39.5)	0.13
Mortality ≤30 d, n (%)	1 (2.4)	1 (2.5)	2 (2.5)	NS
Mortality 31-90 d, n (%)	2 (4.9)	1 (2.5)	3 (3.7)	NS
Mortality 91 d-12 mo, n (%)	1 (2.4)	0	1 (1.2)	NS
Mortality 13-60 mo, n (%)	11 (26.8)	2 (5.0)	13 (16.1)	0.02
Mortality ≥61 mo, n (%)	5 (12.2)	8 (20.0)	13 (16.1)	0.51

DVT = deep vein thrombosis, ORIF = open reduction and internal fixation, PE = pulmonary embolism, UTI = urinary tract infection

Mortality from all causes is shown within 30 days, 90 days, and 12 months.

All causes of mortality during the period of follow-up (mean 64 months, range 0.5 to 199 months) was 48.8% in the nonsurgical group and 30% in the group treated with initial ORIF (*P* = 0.13). However, no differences in mortality within 30 days (2%), 90 days (6.2%), or 1 year (7.4%) were noted. Age when injured was associated with mortality (82.2 versus 64.7, *P* < 0.001).

### Functional Outcomes

Of the 81 patients, only 25 answered the MFA survey (30.9%), with an average score of 25.2, notably worse than an uninjured reference value (9.6, *P* < 0.0001).^14^ The mean time to MFA completion was 59 months (Table 3). Comparison with the total population demonstrated those who completed the MFA to be younger. Only six nonsurgical patients completed a survey. Rates of early complications were comparable between groups; however, late complications and secondary surgeries were more common in the group who completed an MFA survey (28% versus 20%).

**Table 3:** Functional Outcome Scores Measured by MFA for 25 Patients With 26 Fractures

Patient, Injury, and Treatment Features	n (%)	Mean MFA	*P* Value
Male	19 (76)	27.7	0.51
Female	6 (24)	19.3	
Age 60-65 yrs	10 (40)	16.6	
Age 66-70 yrs	5 (20)	23.6	
Age >70 yrs	10 (40)	35.8	0.11^a^
BMI <30 kg/m^2^	11 (44)	31.1	0.27
BMI ≥30 kg/m^2^	14 (56)	20.6	
Current smoker	1 (4.0)	24	
Former smoker	5 (20)	33.6	
Nonsmoker	16 (64)	24.9	0.052^b^
Fracture pattern			
ABC	10 (38.5)	19.3	0.31
APCHT	16 (61.5)	29.6	
Low-energy injury	14 (56)	37.5	0.01
High-energy injury	11 (44)	11.8	
Injuries to other systems	16 (64)	16.8	0.02
Isolated injuries	9 (36)	41.6	
Fracture features			
Hip dislocation	3 (11.5)	30.3	
Marginal impaction	2 (7.7)	47	
ORIF	19 (76)	20.1	0.03
Nonsurgical	6 (24)	43.3	

ABC = associated both column, ACPHT = anterior column posterior hemitransverse, BMI = body mass index, MFA = Musculoskeletal Function Assessment, ORIF = open reduction and internal fixation

Data are shown based on demographic, injury, and social factors.

a *P* = 0.11 for patients aged >70 years versus all others.

b *P* = 0.052 for nonsmokers versus all others.

Trends were observed for worse MFA scores to be associated with older age at the time of injury (35.8 for those greater than age 70 years; *P* = 0.11) and for nonsmokers to have better MFA scores (24.9, *P* = 0.052). No association of MFA scores with sex or BMI was seen. MFA scores were not associated with fracture pattern. However, those sustaining a high-energy injury (motor vehicle collision, fall from height, bicycle accident, or pedestrian versus car) had better outcomes than those with a low-energy mechanism (11.8 versus 37.5, *P* = 0.01). Similarly, patients with isolated injuries fared worse than those with associated injuries to other body systems (41.6 versus 16.8, *P* = 0.02). No baseline MFA scores were obtained; thus, it is unknown whether patients with lower-energy injury, and more often isolated in nature, may have had worse functionality at baseline.

Patients with early complications had similar outcomes than those without (20 versus 27.6, *P* = 0.31), whereas those with late outcomes had worse outcomes than those without (37.6 versus 20.4, *P* = 0.02). Mean MFA for those with PTA was 40.2. Finally, patients who underwent secondary procedures had significantly worse outcomes (45.7 versus 18.7, *P* = 0.007). Patients initially treated nonsurgically had poor outcomes (43.3 versus 20.1, *P* = 0.03), regardless of the presence of early or late complications. However, posttraumatic arthrosis and THA were more frequent after ORIF, suggesting that a subgroup of patients treated surgically who developed PTA and who underwent additional surgeries had very poor outcomes when compared with those without additional surgeries.

## Discussion

Although previous literature has reviewed short- and long-term outcomes after acetabular fracture, this study has focused on a subset of injuries, relatively common in elderly patients after low-energy mechanisms. Our purpose was to determine whether surgical or nonsurgical management of these injuries may be associated with favorable results and outcomes. A secondary objective was to identify factors associated with poor functional outcomes. Notably, in contrast to most proximal femoral fractures, which are extremely common in the aging population, acetabular fractures differ in that immediate weight bearing as tolerated is often not possible after fixation of acetabular fracture, until sufficient healing has occurred.^17,18^

Equal numbers of elderly patients sustaining ACPHT and ABC fractures were treated surgically and nonsurgically. However, high-energy fractures and those with patterns with relative surgical indications such as marginal impaction, femoral head injury, and associated dislocation were more often treated surgically. Despite this, most mechanisms within our patient sample were low energy (71% from low-energy falls). Treatment recommendations may have been influenced by factors including medical comorbidities, body habitus, bone quality, baseline function, fracture pattern, and associated injuries to other body systems. Surgeon experience and preference may also have played a role. One limitation of this study is difficulty in delineating among relative indications for surgery versus nonsurgical management.

Although we were unable to distinguish among patients with similar injuries, baseline conditions, and functional expectations who were treated with either method, to provide a direct comparison, it seems that most patients not only sustained lower-energy injuries but also lacked traditional features of mechanical instability. Thus, one of the strengths of this study is a large group of patients with similar injuries, many of which could be treated with either method.

No differences were noted in early complication rates based on type of treatment, and functional outcomes were not affected by the presence of early complications. Furthermore, mortality within 12 months after injury was not related to type of treatment. This is likely because patients have a similar weight-bearing restriction with either type of treatment. However, PTA was associated with greater likelihood of THA and with worse MFA scores. This is consistent with a large body of previous literature.^20-22^ However, our overall rates of PTA and of THA conversion are favorable, suggesting that many patients in both the initial nonsurgical group and the surgical group had functional results. Patients who demonstrate secondary congruence of the acetabulum with no history of subluxation or dislocation may be treated nonsurgically with low risk of PTA.^10,11^ Overall, the group of patients undergoing ORIF was more likely to develop PTA, which was influenced by initial injury features, particularly history of marginal impaction and/or dislocation.

Better functional outcomes were observed in patients who sustained high-energy injuries and in those with associated injuries to other body systems. Although the entire group likely had more complex articular injuries, and features portending worse prognosis, it seems that these patients may have fared better as a whole group being more likely to undergo ORIF and more likely to recover baseline function. We speculate that the minority of patients with fracture characteristics associated with PTA did not benefit as much from ORIF long term; this represents an interesting group warranting more consideration regarding other initial treatment pathways, perhaps primary THA.

Other factors influenced functional outcome, with trends for older patients and for those consuming tobacco products to have worse MFA scores. This is consistent with other work, and likely due to lower levels of baseline function associated with age and with tobacco use,^11,17,19^ as well as a possibility of altered pain perception, which has been associated with prolonged nicotine intake.^23-25^

Other limitations exist within this study. We are not able to remark on the quality of reduction as a possible contributing factor for PTA and poor outcomes. In addition, there was a low response rate (31%) for completion of the MFA. When comparing outcomes across treatments, it was difficult to draw concrete conclusions due to the small response rate in the nonsurgical group. This was due to either lack of interest, inaccurate phone numbers or address, or death of the patient. The low response rate among the nonsurgical group may also portend a bias toward sampling patients with worse outcomes secondary to lower satisfaction with recovery. Finally, although the MFA survey is a measure of outcome, it fails to consider certain factors such as patient outlook and baseline function. Other measures of functional outcome and quality of life could be analyzed, such as the Short Form-36.^26^ Further study to include more individuals through more years or at more centers would increase validity. Prospective MFA completion during follow-ups would also be informative.

## Conclusion

Most patients sustained low-energy fractures, which may have been amenable to either treatment pattern. Minimally displaced fractures at low risk for symptomatic PTA may be effectively treated nonsurgically, with reasonable functional result. Higher-energy injuries without fracture characteristics portending severe PTA risk seem to benefit from ORIF, as evidenced by good MFA scores. Arthrosis is associated with marginal impaction and with hip dislocation, regardless of the type of treatment. Poor outcomes were associated with PTA, although many of those patients underwent THA for pain relief. Surgical indications include displaced fractures of the weight-bearing articular surface and fracture instability to mitigate PTA. For patients with injury features portending severe PTA risk and/or patients with preexisting arthritis, future investigation into the role of primary or early THA seems warranted.
